# A case report of adenosquamous carcinoma of the esophagogastric junction

**DOI:** 10.1186/s40792-022-01441-6

**Published:** 2022-05-03

**Authors:** Harunari Fukai, Naohiko Koide, Naoe Yanagisawa, Yoshinori Koyama, Nami Kitagawa, Yuu Ogihara, Maki Ohya

**Affiliations:** 1 Department of Surgery, Nagano Prefectural Kiso Hospital, 6613-4 Fukushima, Kiso, Nagano 397-8555 Japan; 2 Department of Internal Medicine, Nagano Prefectural Kiso Hospital, 6613-4 Fukushima, Kiso, Nagano 397-8555 Japan; 3 Department of Clinical Laboratory, Nagano Prefectural Kiso Hospital, 6613-4 Fukushima, Kiso, Nagano 397-8555 Japan; 4 Department of Pathology, Shinshu University School of Medicine, 3-1-1 Asahi, Matsumoto, Nagano 390-8621 Japan

**Keywords:** Adenosquamous carcinoma, Esophagogastric junction, Submucosal tumor-like

## Abstract

**Background:**

Many types of tumors can arise in the esophagogastric junction (EGJ). Squamous cell carcinoma (SCC) arising from the esophageal epithelia, adenocarcinoma arising from the gastric mucosa, or Barrett’s esophageal mucosa are frequently observed in the EGJ. However, adenosquamous carcinoma (ASC) has been rarely observed in this area. We herein report a rare case of ASC of the EGJ.

**Case presentation:**

An 81-year-old man visited our hospital complaining of dysphagia. Esophagogastroduodenoscopy detected an elevated tumor in the gastric cardia. Biopsy specimens taken from the tumor showed SCC. Computed tomography revealed a tumor located in the EGJ and node metastases surrounding the EGJ. The tumor was diagnosed as SCC, overhanging in the stomach, of the EGJ. The patient underwent a proximal gastrectomy with a lower esophagectomy and node dissection for the metastases surrounding the EGJ, and esophagogastrostomy in the lower mediastinum. Histopathologic examination showed the tumor consisted of SCC and adenocarcinoma. The adenocarcinoma consisted of nests scattered in the SCC. We observed adenocarcinoma component in 35% of the tumor and epithelial spread of SCC in the lower esophagus. Thus, we diagnosed the tumor as ASC of the EGJ. Eight metastatic nodes were dissected; both SCC and adenocarcinoma were observed in seven.

**Conclusions:**

In the present case, SCC may be originated from the squamous epithelia of the lower esophagus and grew into the stomach, and the adenocarcinoma may have differentiated from SCC through the infiltration.

## Background

Tumors of the esophagogastric junction (EGJ) have many issues, including diagnosis and treatment [1]. Squamous cell carcinoma (SCC) arising from the esophageal epithelia, adenocarcinoma arising from the gastric mucosa, or Barrett’s esophageal mucosa are frequently observed in the EGJ. However, adenosquamous carcinoma (ASC) has been rarely observed in this area. We herein report a rare case of histopathologically diagnosed ASC in the EGJ, and we discuss the infiltration and differentiation of the tumor.

## Case presentation

**Fig. 1:**
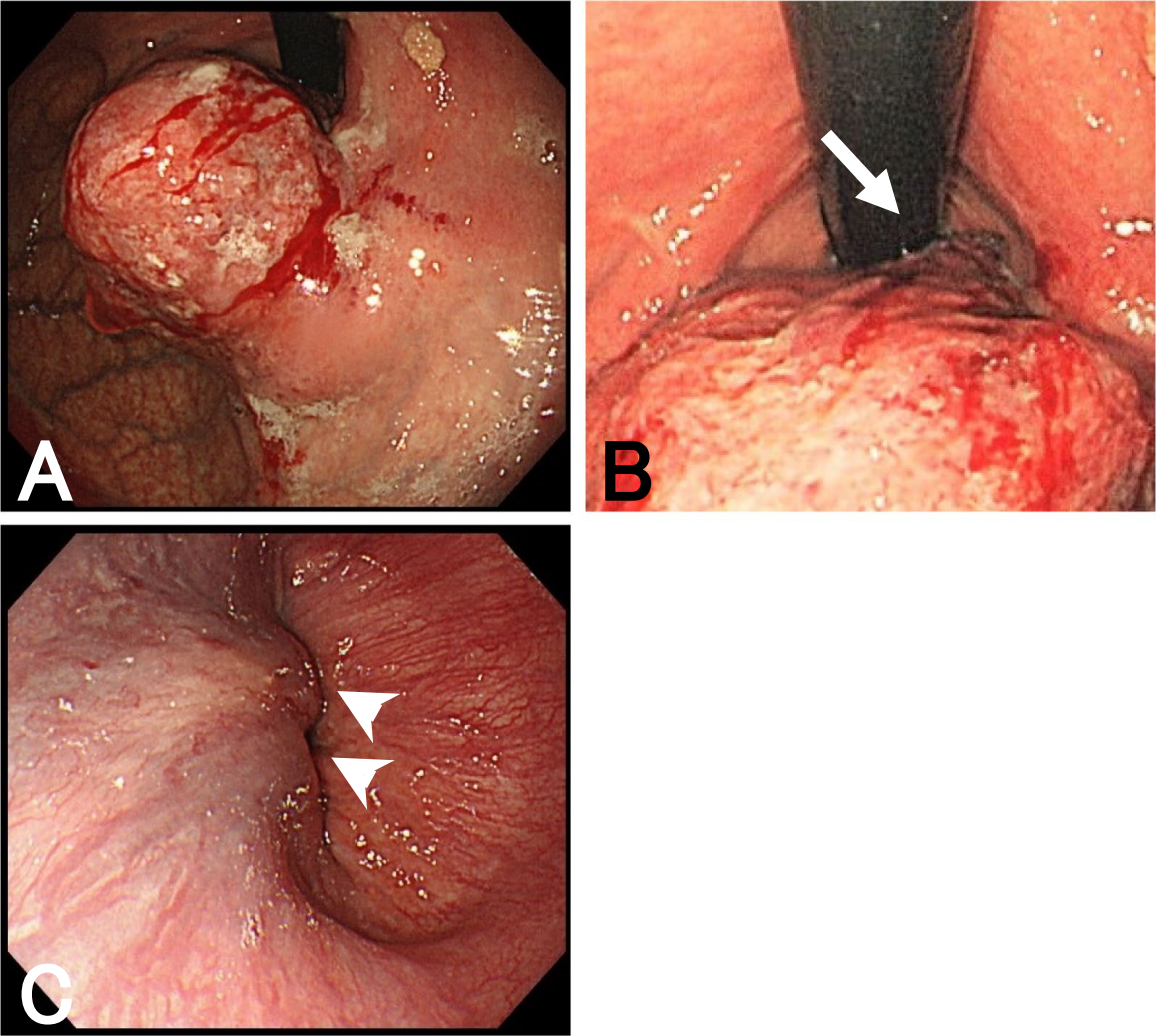
Esophagogastroduodenoscopy findings. **A** An elevated lesion is found in the EGJ. The tumor is easy to bleed, and its demarcation is ill-defined because of submucosal invasion. **B** The tumor overhangs in the gastric cardia, but continues into the squamocolumnar junction (arrow). **C** In the lower esophagus, the tumor invades in the esophageal submucosa (arrowheads). The lumen of the EGJ is intact

**Fig. 2:**
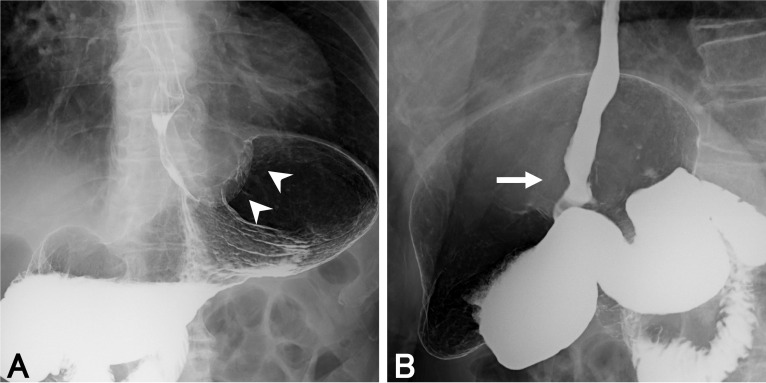
Findings of barium meal study. **A** An elevated lesion is detected in the gastric cardia (arrowheads). **B** The tumor invades the lower esophagus approximately 15 mm from the EGJ with stenosis (arrow)

**Fig. 3:**
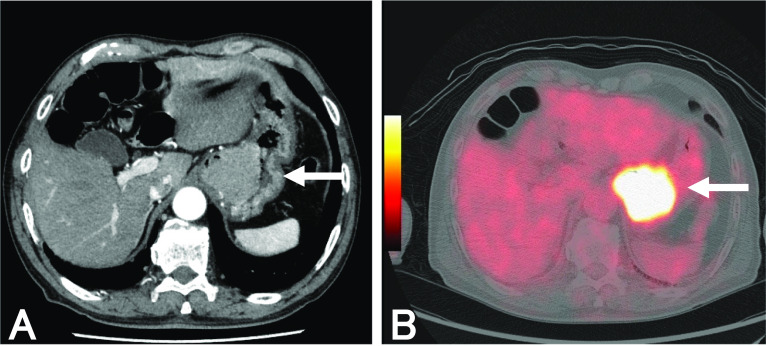
Image findings. **A** Computed tomography. Computed tomography reveals a mass, 50 mm in diameter, in the EGJ (arrow). **B** ^18^F-FDG-positron emission tomography. In 18F-FDG-positron emission tomography, SUV max of FDG accumulation is 19.9 in the tumor (arrow)

An 81-year-old man visited our hospital complaining of dysphagia. The patient received an upper lobectomy of the right lung for lymphoma and additional chemotherapy 20 years ago. Esophagogastroduodenoscopy detected an elevated tumor in the EGJ (Fig. 1A). The tumor was mainly located in the stomach, but it continued into the squamocolumnar junction (Fig. 1B). We found a submucosal invasion of the tumor in the lower esophagus (Fig. 1C). Biopsy specimens taken from the tumor showed SCC histopathologically. Laboratory examination showed that the serum level of SCC was elevated (25.2 ng/ml, normal range < 2.5 ng/ml), whereas the serum levels of CEA and CA19-9 were in normal range (3.4 ng/ml, < 5 ng/ml and 7 U/ml, < 37 U/ml, respectively). Barium meal study showed an elevated lesion in the gastric cardia (Fig. 2A), which had invaded the lower esophagus approximately 15 mm from the EGJ (Fig. 2B). Computed tomography showed a tumor, 50 mm in size, located in the EGJ and several lymph nodes surrounding the EGJ were swollen (Fig. 3A). No mediastinal node metastasis was found, and hepatic and lung metastasis were not detected. In ^18^F-fluorodeoxyglucose (FDG)-positron emission tomography, the standardized uptake value (SUV) max of FDG accumulation was 19.9 in the tumor (Fig. 3B). Respiratory function test showed an obstructive disorder because of previous smoking. Although the serum level of HbA1c was 6.3%, fasting blood glucose was 123 mg/dl. He was administered drugs for hypertension.

**Fig. 4:**
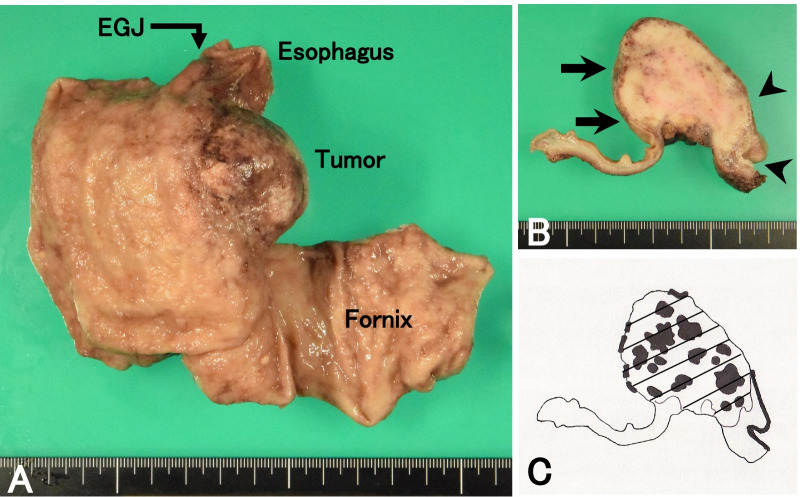
Macroscopic findings. **A** Macroscopic findings of the resected specimen. An elevated tumor is mainly located in the gastric cardia. **B** The cut surface of the tumor. The tumor is covered with the normal squamous mucosa (arrowheads) and the gastric mucosa (arrows). **C** A schema of the cut surface. Black areas show the adenocarcinoma components, while diagonal area shows the SCC components. The adenocarcinoma component is located diffusely as a focal area in the tumor and occupies 35% of the tumor, while the SCC component is located in the surface to the deep layer of the tumor.

**Fig. 5:**
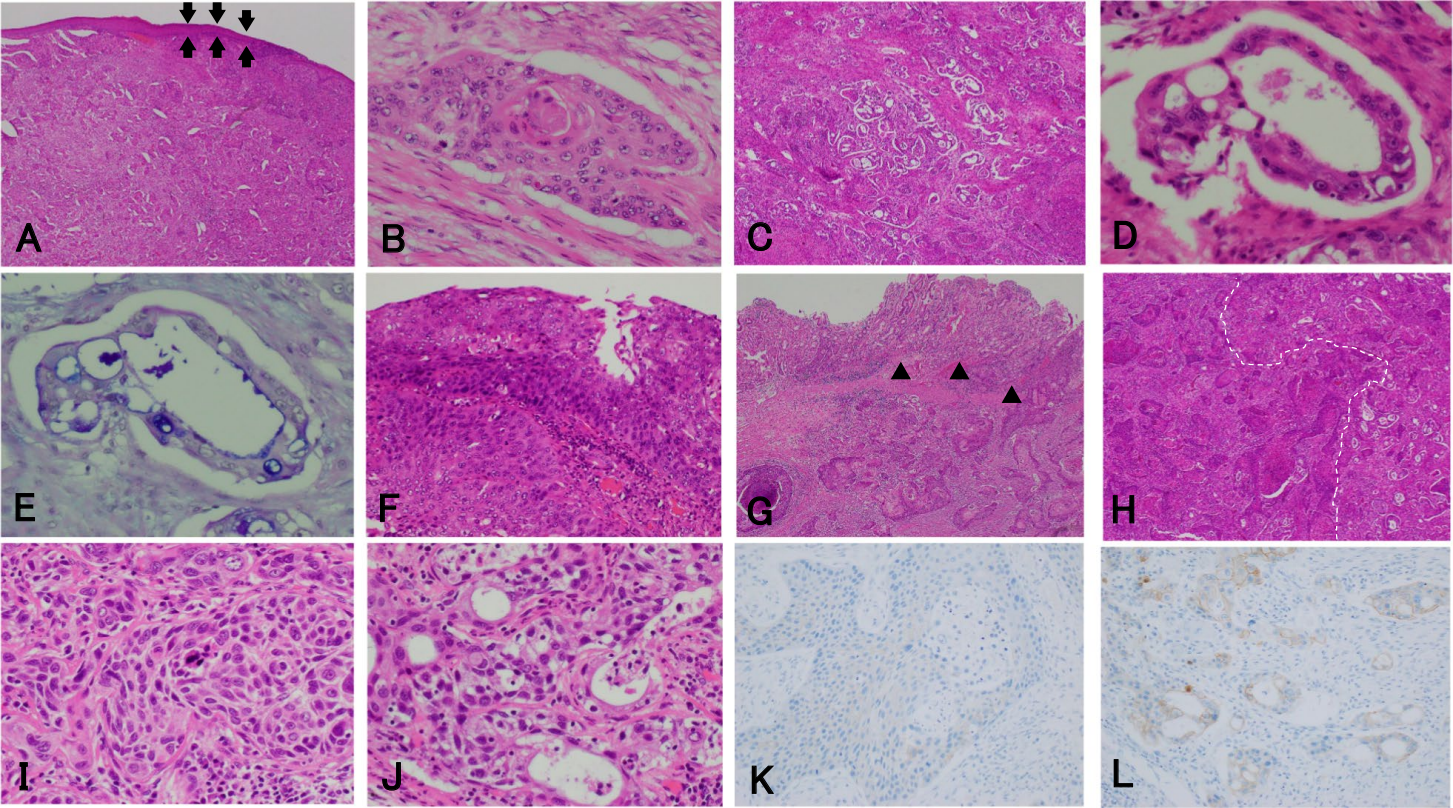
Histopathologic findings. **A** Low-power magnification of SCC in hematoxylin and eosin staining (HE). SCC, covered with the normal squamous epithelium, is infiltrated diffusely in the tumor (arrows). **B** High-power magnification of SCC (HE). SCC is moderately differentiated. **C** Low-power magnification of adenocarcinoma (HE). Scattered adenocarcinoma nests are observed in the tumor. **D** High-power magnification of adenocarcinoma (HE). Adenocarcinoma is well-differentiated. **E** AB-PAS staining shows blue adenocarcinoma cells. **F** Superficial SCC in the squamous epithelium of the lower esophagus. **G** Distal part of SCC is covered with the normal columnar epithelium (arrowheads). **H** Several transition parts between SCC and adenocarcinoma components are observed in the primary tumor. The left panel shows SCC (left side on the dashed line) and the right panel shows the adenocarcinoma component (right side on the dashed line). **I** The SCC component is observed in the No. 11p nodes dissected. **J** The adenocarcinoma component is observed in the No. 11p nodes synchronously. **K** No reaction for HER2 is immunohistochemically observed in SCC cells. **L** A positive reaction for HER2 is observed in adenocarcinoma cells, the staining intensity is judged to be 2+

We diagnosed the tumor as SCC, overhanging in the stomach, of the EGJ before surgery. Considering the frailty due to the influence of neoadjuvant chemotherapy, we selected surgery because of the elderly. The patient underwent a proximal gastrectomy combined with lower esophagectomy and node dissection surrounding the EGJ, the celiac trunk with its branches, and the lower esophagus. The lower esophagectomy and dissection surrounding the lower esophagus were made by a median incision of the diaphragm from the hiatus. Proximal margin of the esophagus 3 cm apart from the EGJ was taken. During the operation, washing cytology using saline showed no cancer cells. After removal of the tumor, frozen sections were made and no cancer invasion was confirmed in both the proximal margin of the esophagus and the distal margin of the stomach. We performed esophagogastrostomy using a circular stapling system in the mediastinum. Macroscopic findings showed a tumor was located in the EGJ (Fig. 4A), and the tumor showed a submucosal progression (Fig. 4B). The postoperative course was uneventful. Histopathologic examination showed the tumor consisted of SCC (Fig. 5A, B) and adenocarcinoma (Fig. 5C–E). We observed SCC continuously in the squamous epithelia of the lower esophagus (Fig. 5F). *Helicobacter pylori* was not microscopically detected. In the distal part of the tumor, SCC was covered with the normal columnar mucosa (Fig. 5G). Several nests of adenocarcinoma were scattered in the tumor and occupied 35% of the tumor; however, the SCC component was located in the surface to the deep layer of the tumor (Fig. 4C). We observed a transition between SCC and adenocarcinoma (Fig. 5H). Node metastasis was observed in eight of dissected 32 nodes. Seven of the eight nodes showed both metastases of SCC and adenocarcinoma (Fig. 5I, J), and a node in the right area of the gastric cardia showed metastasis of SCC (Table 1). No reaction for human epidermal growth factor receptor-2 protein (HER2) was immunohistochemically observed in SCC cells. A weak positive reaction for HER2 was observed in adenocarcinoma cells, and the staining intensity was judged to be 2+ (Fig. 5K, L). Thus, the tumor was diagnosed as ASC of the EGJ, and the TNM classification (UICC 8th edition) was pT3 pN3 M0, pStage IVa. The patient received adjuvant chemotherapy using S-1 orally because of the elderly with the frailty, and was well without recurrence 6 months after surgery.

**Table 1:** SCC and adenocarcinoma in node metastasis

Node position*	No. of dissected nodes	No. of metastatic nodes	SCC	Adenocarcinoma
Rt. side of gastric cardia (No. 1)	2	2	(+)	(−)
Lesser curvature (No. 3a)	1	1	(+)	(+)
Lt. gastric artery (No. 7)	6	3	(+)	(+)
Splenic artery (No. 11p)	2	2	(+)	(+)

*SCC* squamous cell carcinoma

*Node numbers are described according to the Japanese Classification of Gastric cancer (15th ed.) [2]

## Discussion

ASC is exceedingly rare in the EGJ. We identified three important clinical issues in the present case: (1) ASC was considered to be originated from the squamous epithelia; (2) ASC of the EGJ can present as a submucosal tumor-like mass in the EGJ, and (3) both SCC and adenocarcinoma were metastasized in regional nodes.

No data of frequency of ASC in the EGJ is available. ASC is less frequently found in the esophagus. Previous studies reported it to be 0.37–1% of esophageal carcinoma in Western countries [3–6], and 0.6–1.0% in Japan [7, 8]. In gastric cancer, previous studies reported it to be less than 1% in Western countries [9], and 0.14–1.3% in Japan [10, 11].

Various theories regarding the origin of ASC have been suggested in esophageal ASC. Pascal and Clearfield [12] reported that ASC in the esophagus arises from the esophageal gland cells or the ductal cells. As the epithelium and submucosal glands are derived from the foregut during embryogenesis, adenocarcinoma has the potential to transform into SCC. Other authors [6, 8, 13–16] considered that ASC arises from the mucosa, where it develops into SCC firstly and then the glandular cells differentiate into ASC. Furthermore, a collision concept was proposed, in which ASC may come from two individual stem cells that independently and simultaneously undergo malignant transformation [17, 18]. In esophageal ASC, most of the adenocarcinoma component is located at an invasive site. On the other hand, the SCC component is located in the superficial epithelial region adjacent to the adenocarcinoma component.

Five hypotheses have been proposed in gastric ASC: (1) squamous metaplastic transformation of adenocarcinoma [19–22]; (2) oncogenic transformation of the ectopic squamous epithelium [23]; (3) oncogenic transformation of the metaplastic non-neoplastic squamous cells [24], (4) collision of concurrent adenocarcinoma and SCC [25], and (5) differentiation of multipotential undifferentiated cancer cells toward both the glandular and squamous cells [26, 27]. In gastric ASC, most of the SCC component was located at an invasive site. In contrast, the adenocarcinoma component was located in the superficial mucosal region adjacent to the SCC component. Thus, many authors now believe that the SCC component results from metaplastic change of the adenocarcinoma component in gastric ASC. In the present case, the tumor was considered to be originated from the esophageal squamous epithelium, where it developed into SCC, and then SCC differentiated to adenocarcinoma sporadically. As the tumor continued into the squamous epithelium, it was mainly composed of the SCC component, and it was covered with the normal squamous epithelium and scattered SCC in the EGJ histopathologically.

It is interesting to note that the tumor was located in the stomach mainly with submucosal invasion, even though the origin of the tumor was considered to be the esophageal epithelium histopathologically. Kuwano et al. [28] proposed that there are four patterns of invasion of the stomach in esophageal SCC; however, these patterns did not include invasion of the gastric submucosa. Furthermore, Iriguchi et al. [29] reported a case of SCC located in the submucosa of the gastric cardia, developing from the esophageal mucosa of the EGJ. According to previous studies, SCC of the EGJ invading the gastric submucosa is rare. In contrast, special pathological types of esophageal carcinoma, such as ASC, may show a submucosal tumor-like form. Matsuda et al. [30] reported that three out of five cases of ASC in the esophagus showed subepithelial growth as a submucosal tumor-like form. Based on these findings, it is difficult for SCC to grow under the submucosa; however, it is easy for ASC to grow under the submucosa in the esophagus. The mechanism of invading the submucosa in the esophagus is that the cancer that occurs from the parabasal layer of the epithelium shows a downward growth, cancer arises from the esophageal gland cells or the ductal cells, and cancer arises from the esophageal cardiac glands [31]. The tumor may arise from the esophageal gland cell, the ductal cells, or the cardiac glands; however, we were unable to prove this histopathologically in the present case.

Node metastasis in ASC of the EGJ also remains poorly understood. Previous studies found that 33% of patients with esophageal ASC had node metastasis [32, 33]. Among them, SCC component accounted for 60.9–85.7%, both components accounted for 14.3–26.1%, and adenocarcinoma component accounted for 0–8.7%. In contrast, 70.2–83% of patients with gastric ASC had node metastasis [10, 11]. Among them, adenocarcinoma component, both, and SCC component accounted for 63%, 26%, and 11%, respectively. In the present case, seven of the eight nodes showed both metastases of SCC and adenocarcinoma, and a node in the right area of the gastric cardia showed metastasis of SCC only. We considered that many metastases had both components due to the volume of the tumor. Thus, esophageal ASC showed node metastasis from the SCC component, whereas gastric ASC showed metastasis from the adenocarcinoma component. In the present study, ASC was found in the EGJ; thus, metastasis and/or recurrence from both components should be considered in the future. In addition, HER2 was negative by fluorescence in situ hybridization; however, immunohistochemistry was 2+ in adenocarcinoma cells. When HER2 was positive, trastuzumab may be indicated for treatment of ASC of the EGJ.

## Conclusions

We report a rare case of ASC showing specific growing of the EGJ and discussed its growth pattern.

## Data Availability

Not applicable.

## Abbreviations

EGJ: Esophagogastric junction
SCC: Squamous cell carcinoma
ASC: Adenosquamous carcinoma
FDG: ^18^F-fluorodeoxyglucose
SUV: Standardized uptake value
HER2: Human epidermal growth factor receptor-2 protein
HE: Hematoxylin and eosin staining
